# Letter to the Editor Re: Nissensohn M. et al.; *Nutrients* 2016, *8*, 232

**DOI:** 10.3390/nu8080453

**Published:** 2016-07-27

**Authors:** Cíntia Ferreira-Pêgo, Nancy Babio, Jordi Salas-Salvadó

**Affiliations:** 1 Human Nutrition Unit, Biochemistry Biotechnology Department, Faculty of Medicine and Health Sciences, Universitat Rovira i Virgili, IISPV (Institut d’Investigació Sanitària Pere Virgili), Hospital Universitari de Sant Joan de Reus. C/Sant Llorenç, 21, Reus 43201, Spain; cintia.ferreira@iispv.cat (C.F.-P.); nancy.babio@urv.cat (N.B.); 2 CIBEROBN (Centro de Investigación Biomédica en Red Fisiopatología de la Obesidad y Nutrición), Instituto de Salud Carlos III, Av. Monforte de Lemos, 3-5, Pabellón 11, Planta 0, Madrid 28029, Spain

Dear Editor,

We read with interest the recently published original article entitled “Beverage Consumption Habits and Association with Total Water and Energy Intakes in the Spanish Population: Findings of the ANIBES Study” by Nissensohn et al. [1] in *Nutrients*.

We agree with the authors that there is a clear need for more information about total fluid and different types of beverages intake and further epidemiological studies using this information should be performed. However, we note in the Introduction the following sentences: “Furthermore, total water intake (TWI) data of Spain are scarce. There are no recent epidemiological studies that focus exclusively on beverages intake. […], we are unaware of other research investigating beverage intake among the Spanish population.”

We would like to point out that, in 2014, our group in collaboration with other authors published a cross-sectional description of the fluid intake habits of the Spanish population including 1262 adults [2] and 238 children and adolescents aged 3–17 years [3]. These articles described exclusively the beverage consumption of the Spanish population and analyzed it according sex, age ranges and time of day. We also compared our results on fluid and beverage consumption with EFSA water adequate intakes and WHO free sugar recommendations [4,5].

We subsequently related this fluid intake data to Mediterranean diet adherence and physical exercise practice [6]. We observed that adult individuals presenting a higher adherence to the Mediterranean diet and those practicing more physical exercise also presented a healthy beverages consumption pattern, showing moderate consumption of wine and milk and derivatives, high consumption of water and lower intake of sugar-sweetened beverages.

Finally, in 2015, we compared the Spanish fluid and beverage intake with 12 other countries worldwide using the same methodological approach [7,8]. In these two publications, we compared the fluid consumption with the EFSA total fluid intake and WHO free-sugar recommendations, in order to assess the percentage of population not complying with these recommended values.

The conclusions of Nissensohn et al. are very similar of those of our research group: a high proportion of the population in Spain are not complying with the EFSA adequate intake values.

## References

[1] Nissensohn M., Sánchez-Villegas A., Ortega R.M., Aranceta-Bartrina J., Gil Á., González-Gross M., Varela-Moreiras G., Serra-Majem L. (2016). Beverage Consumption Habits and Association with Total Water and Energy Intakes in the Spanish Population: Findings of the ANIBES Study. Nutrients.

[2] Ferreira-Pêgo C., Babio N., Fenández-Alvira J.M., Iglesia I., Moreno L.A. (2014). Fluid intake from beverages in Spanish adults: Cross-sectional study. Nutr. Hosp..

[3] Fenández-Alvira J.M., Iglesia I., Ferreira-Pêgo C., Babio N., Salas-Salvadó J., Moreno L.A. (2014). Fluid intake in Spanish children and adolescents; a cross-sectional study. Nutr. Hosp..

[4] Agostoni C., Bresson J., Fairweather-Tait S. (2010). Scientific opinion on dietary reference values for water. EFSA J..

[5] World Health Organization (2015). Guideline: Sugars Intake for Adults and Children.

[6] Ferreira-Pêgo C., Babio N., Salas-Salvadó J. (2015). A higher Mediterranean diet adherence and exercise practice are associated with a healthier drinking profile in a healthy Spanish adult population. Eur. J. Nutr..

[7] Guelinckx I., Ferreira-Pêgo C., Moreno L.A., Kavouras S.A., Gandy J., Martinez H., Bardosono S., Abdollahi M., Nasseri E., Jarosz A. (2015). Intake of water and different beverages in adults across 13 countries. Eur. J. Nutr..

[8] Ferreira-Pêgo C., Guelinckx I., Moreno L.A., Kavouras S.A., Gandy J., Martinez H., Bardosono S., Abdollahi M., Nasseri E., Jarosz A. (2015). Total fluid intake and its determinants: Cross-sectional surveys among adults in 13 countries worldwide. Eur. J. Nutr..

